# Similar but different: A qualitative study of occupational therapists' transformational experiences during practice education

**DOI:** 10.1111/1440-1630.70028

**Published:** 2025-06-08

**Authors:** Anne‐Maree Caine, Olympia Nicolson, Matthew Molineux

**Affiliations:** ^1^ Griffith University Brisbane Australia

**Keywords:** experiential learning, occupational therapy, placement, practice education, transformational experiences

## Abstract

**Introduction:**

Practice education is a key component in the education of occupational therapists, and yet there is limited understanding of the long‐term impact of practice education experiences. This study explored transformational experiences on practice education to better understand their long‐term impact. Transformational experiences are those that cause a profound and fundamental change in a person's view of themselves and/or the profession. The aim of this study was to explore the transformational experiences, both positive and negative, of occupational therapists while they were on practice education as students.

**Methods:**

A phenomenological research design was used to understand the lived experiences of occupational therapists who had undergone transformational experiences while students on practice education. Data were collected using semi‐structured interviews from 11 participants who had graduated at least 5 years prior to this study. Data analysis was completed using interpretative phenomenological analysis.

**Consumer and Community Involvement:**

No consumers were involved in this study.

**Findings:**

An overarching theme of *similar but different* was identified, which recognised that while each experience was unique there were some shared elements: the experiences were challenging, emotionally laden, took time to process and had long‐term impacts. Positive and negative experiences were precipitated by a challenge of some kind. Participants experienced a wide spectrum of emotions at the time of the event and for some the emotional response lasted long afterwards. It took time and reflection to process their experiences, and they often required help to develop insights into the experience and its impact. Long‐term impacts were experienced personally and professionally.

**Conclusion:**

This study demonstrated that students have transformational experiences on practice education. These can be ordinary or extraordinary and therefore unique to each student, making them difficult to predict. The findings highlight the value of practice educators and university educators checking in with students to support them to reflect on and process transformational experiences.

**PLAIN LANGUAGE SUMMARY:**

Placements are an important part of educating student occupational therapists. We do not know much about how experiences on placement affect students over time. This study looked at how certain experiences during placements changed students over time. We called these ‘transformational experiences’. That means any event, big or small, that had a strong a lasting effect on the person. We spoke with 11 occupational therapists who had finished their studies at least 5 years ago. They chose to take part because they remembered a powerful experience during their placement. The stories they shared were all different, but they had some things in common. These experiences were hard, emotional and took time to understand. They also had long‐term effects. We found that transformational experiences can be both ordinary and unusual. This makes them hard to predict.

**Key Points for Occupational Therapy**

- Some students have transformational placement experiences that have long‐term impacts.
- Practice educators may benefit from ongoing education and support regarding the long‐term impact practice education experiences can have on students personally and professionally.
- Students, university staff and practice educators should collaborate to identify transformational experiences and support all those involved.

## 1 INTRODUCTION

Practice education (PE) is a key component in the education of occupational therapists (World Federation of Occupational Therapists [WFOT], 2016). PE experiences are a form of experiential learning used to develop students' knowledge, skills and attitudes so they can practice with various people in a range of contexts (WFOT, 2016). The resources and effort committed to PE by students, practice educators, placement providers and universities are significant, so it is important to ensure these experiences are constructive and beneficial for emerging occupational therapists (WFOT, 2016). Further, the extent to which these formative experiences may be transformational, that is, the way they impact on occupational therapists over the medium and long‐term, needs to be understood in order to inform preparation and supervision practices. For the purpose of this study, the term ‘transformational experience’ was adapted from Mezirow's (1991) concept of transformational learning and is used here to describe an experience that causes a fundamental and profound change in one's worldview and behaviour.

Research on occupational therapy PE primarily focuses on university curriculum and students' perceptions of these experiences (Roberts et al., 2015). WFOT (2016) acknowledges that students, new graduates, practice educators and university staff provide valuable feedback on placement, which is used to inform placement planning, preparation and provision. However, longer‐term impacts of placement are often overlooked because evaluation often does not look beyond short‐term impacts and outcomes (Roberts et al., 2015).

There is very limited literature that focuses specifically on transformational experiences in PE. However, such experiences have been documented indirectly, with some studies hypothesising long‐term impacts. Research into international and rural placements is one area where this has occurred. Participants have reported significant changes in their behaviour and worldview, after becoming immersed in unfamiliar cultures and environments (Crawford et al., 2017; Davies et al., 2017; Haro et al., 2014; Markewitz, 1992; Sim & Mackenzie, 2016; van Rensburg & Du Toit, 2016). Participants in Davies et al.'s (2017) Australian interpretive phenomenology study of nine participants who completed an international occupational therapy placement found experiences were intense and resulted in personal and professional development beyond that which would have occurred on a typical domestic placement in Australia. Participants learned much about themselves personally, developed cultural understanding and appreciation and were assisted to prepare for the transition from student to new graduate (Davies et al., 2017). Markewitz (1992) presented a narrative of her domestic occupational therapy placement, and in that identified that it was the isolation of a rural Alaskan town that was the biggest factor that affected her professional development. Her experience taught her about relating and adjusting to the culture of others, something she reported she would carry with her throughout her career (Markewitz, 1992). A mixed methods study of 25 student occupational therapists in the United Kingdom on role emerging placements revealed that while students had positive and negative experiences there were positive impacts on the development of professional identity and resilience (Cade, 2023), which would likely impact into the future.

Transformational experiences were also documented in personal narratives by student occupational therapists sharing significant placement experiences and their impacts (Archer, 1999; Markewitz, 1992; Warne & Hoppes, 2009). In Archer's (1999, p. 73) personal account he described his ‘very protracted and painful’ journey as a mature aged student with learning disabilities, whose requests for accommodations were denied by his practice educators and university to the point where he was forced to leave the profession. Archer (1999, p. 73), who eventually completed his education and became an occupational therapist, closed by stating he felt he was ‘vastly better equipped to enter [the] profession’ due to his experience, as he not only understood his rights and responsibilities in relation to disability accommodations but had gained insight into his clients' emotional processes. An autoethnography by Warne and Hoppes (2009) explored a student occupational therapist's first encounter with death whilst on placement, and the meanings, challenges and lessons end‐of‐life care held for her. This highly emotional experience left the author with a changed worldview, as her client taught her to ‘slow down and appreciate the vivacity around [her]’, prompting her to reprioritise what she considered important in life (Warne & Hoppes, 2009, p. 314).

Lastly, negative placement experiences already documented in research (Lew et al., 2007), including workplace violence against student occupational therapists and social workers (Gair & Thomas, 2008) and bullying (Boniface & Drynan, 2024), could be viewed as transformational experiences. Examples included bullying by practice educators, being placed in unsafe situations and abuse with the threat of violence due to students' cultural background, age, disability or sexual orientation (Gair & Thomas, 2008). Lew et al.’s (2007) qualitative study of 13 participants is one study that has explicitly examined the long‐term impacts (up to 10 years after the placement) of negative placement experiences from the perspective of occupational therapists. Participants reported impacts on their own behaviour as practice educators, as well as increased self‐reliance and resilience. In Boniface and Drynan's (2024) study of bullying there were similar long term impacts including emotional distress but also strengthened resilience. The majority of participants in Lew et al.’s (2007) study reported that, as graduates, they chose a practice setting different to where they had their negative experience. The impact of placement experiences on preference for work setting after graduation has been long established in occupational therapy literature (Chiang et al., 2013; Christie et al., 1985; Crowe & Mackenzie, 2002; Keller & Wilson, 2011).

While there is some literature that considers the transformational experiences that take place on placements, it has tended to focus on short term impacts. Given the time and resources devoted to practice education, a deeper understanding of the longer‐term impact of transformational placement experiences is warranted. Therefore, the aim of this study was to explore the transformational experiences occupational therapists had while on placement as students. Specifically, the focus of this research was on those atypical experiences, which had impacts beyond those that would typically be expected from PE. The objectives were to:

1. understand what placement experiences, both positive and negative, occupational therapists found transformational;
2. understand the long‐term impact of the transformational experience on occupational therapists; and
3. understand the features of a transformational experience.

## 2 METHODS

### 2.1 Research design

This qualitative study adopted a social‐constructivist theoretical lens with a phenomenological design (Creswell, 2013), to understand the lived experiences of occupational therapists while they were on placement. This study explored the phenomenon of transformational experiences and their impacts, from the perspective of participants (Depoy & Gitlin, 2005), rather than their achievement of specific learning outcomes or skill development, which are frequently the focus of occupational therapy PE literature. Ethics approval was obtained from the Griffith University Human Ethics Committee (GU Ref: 2021/866).

### 2.2 Positionality

The authors of this paper, at the time the research was conducted, included a student occupational therapist and two occupational therapy academics. The student was completing PE during the final stages of the research. One academic had over 16 years of experience working with and supporting students and occupational therapists during PE. The other academic had experience managing occupational therapy programs and developing PE opportunities. Both academics undertook PE as part of their entry‐level education and have been practice educators in traditional and non‐traditional PE experiences. None of the authors had PE experiences as students that would be considered transformational, but the two academics have supported students and practice educators through such experiences.

### 2.3 Participants

Convenience and snowball sampling was employed to recruit participants (Liamputtong, 2012). Participants were recruited using advertisements on social media, the Occupational Therapy Australia website, and emails to practice educator and professional networks. The inclusion criteria were occupational therapists who had graduated from a WFOT approved education program at least 5 years prior to the start of the study and could participate in interviews in English. The decision to recruit experienced occupational therapists rather than students or new graduates was to capture the medium‐ and longer‐term impacts of the transformational experiences. Prospective participants were sent a participant information sheet and consent form to complete prior to data collection.

### 2.4 Data collection

Qualitative data was collected through semi‐structured interviews. An interview schedule (see Appendix A) was developed and pilot tested by each interviewer. Amendments were made to the interview schedule based on feedback and reflection. Six interviews took place online via Microsoft Teams and seven in‐person, depending on each participant's location and preferences. Interviews were conducted by one of the researchers (AMC or MM). Interviews lasted 30–60 min and were audio and/or video recorded. Participants were asked demographic questions at the beginning of the interview. Demographic information included when and from where participants graduated, time practising as an occupational therapist, and practice settings they had worked in since graduating. Interviewers then asked participants to share their transformational experience, with interviewers being guided by the interview schedule and participants' narrative to comprehensively explore the experience.

### 2.5 Data analysis

Interview recordings were transcribed verbatim by transcription services or using the Microsoft Teams transcription feature. Transcripts were checked against recordings for accuracy, and personal information was removed and pseudonyms assigned (Tuckett, 2005). Data were thematically analysed using the interpretative phenomenological analysis (IPA) approach (Smith et al., 2009). This approach was appropriate to explore how participants made sense of their experience and incorporated the expertise of the researchers during the analysis and interpretation process (Dean et al., 2006). The research team followed a process of (1) reading a transcript multiple times; (2) initial noting of how participants viewed and understood the experience; (3) identifying emergent themes and connections; (4) abstracting and integrating themes; (5) beginning the process again with the next transcript; and (6) looking for patterns or connections across cases (Smith et al., 2009). To promote trustworthiness, stage 2 was completed independently by two researchers and compared to ensure consistency. Stage 3 was completed independently by the first author and then discussed and refined in team meetings. Stages 4 and 6 were conducted collectively by all three authors. Themes were developed and refined through iterative discussions during regular team meetings, before being drafted and refined for the last time by the first author.

## 3 FINDINGS

Eleven participants were interviewed and each shared one to three transformational experiences, resulting in 16 experiences (see Table 1). Seven of the experiences were single events that occurred during a placement, seven were the entirety of the placement experience. Two experiences appeared to involve both forms of experiences, that is, a transformational event within a transformational placement experience.

**TABLE 1:** Summary of participants and experiences.

Participant (pseudonym)	Years since graduation (years)	Summary of transformational experiences
Isla	43	Placed at an institution for children with complex disabilities and met young girl who lived with untreated hydrocephaly. She was shocked at the reality that this girl was unable to support the weight of her head herself and spent her days laying on a beanbag, with little quality of life.
Justine	5	Placed at a student led clinic and learned the value of driving her own learning because she realised that she was making greater progress than her student peer who was on placement with her.
		Encountered her client who had absconded from the hospital in the community while out with her partner and had to call her practice educator to ensure her client's safety. It highlighted the importance of having strategies for encounters with clients in the community.
Carly	5	Experienced bullying by her practice educator due to her age, including comments about her hair, young age and where she was from. The experience reinforced her feeling as an outsider and motivated her to perform the role of practice educator differently.
Amahle	19	Grew up in apartheid South Africa and experienced prejudice at university, including from academic staff. Her white practice educator validated her lived experience as a person of colour, and the importance of its role within her practice, an experience she revisits when diversity in the profession is discussed.
Belinda	41	Assisted a man who had experienced a stroke to put on his shoe and worried what she was doing was wrong until her practice educator provided positive feedback. She recognised the impact occupational therapy could have, and as a practice educator is aware of the power differential between educator and student.
		Placed at a residential institution for older adults and those with complex disabilities and was asked to assist her client to shower. She was shocked to learn this involved ‘hosing down’ her client in a communal shower with no dividers, at the same time as the other clients. It prompted her to work in a mental health setting and to practice differently.
Chris	18	Exited an initial interview too quickly. His practice educator sent him back into the room and said not to come back out until the hour was over. Recognising the impact of such a power differential influenced his future practice as an educator.
		Failed his mental health placement. He was assigned the task of enabling his client to shower, even though he had not showered for many years. He learned to empathise with others when they experienced a failure to achieve something.
		Accompanied his client to a doctor's appointment and witnessed the doctor possibly misdiagnose his client. It reduced his own trust in health professionals and taught him the importance of listening to clients.
Delia	22	She questioned the meaningfulness of a pottery group for her clients on a mental health ward who were typically working‐class men. Her practice educator stated pottery was an activity used to keep the clients busy. She became determined to never substitute meaningless activity for occupation in practice.
Ellie	5	Entrusted to set up an in‐house splinting clinic. She valued the responsibility her practice educator gave to her and her student peer. It built confidence and trust in her own ability to complete challenging work tasks.
Frankie	24	Practice educator organised for her to observe around 50 different occupational therapists within the metropolitan hospital. her understanding of the scope and impact of occupational therapy was broadened exponentially.
		Practice educator was not supportive of her doing occupation‐based interventions within the stroke rehabilitation setting. She decided she would never work in stroke rehabilitation and drove her passion for occupation‐centred practice.
Georgia	7	Completed a simple meal preparation assessment and concluded her client was unsafe for discharge the next day. She presented her professional judgement to the registrar who wanted to send the client home. She recognised the importance of speaking up and advocating for clients, for clinicians and students.
Hayden	14	Bullied by his practice educators and failed his placement, which challenged his view of the profession as one that is caring. University support and Hayden's subsequent placement restored his view the profession.

Most participants (n = 8) were graduates from one of five Australian universities. Others graduated from universities in South Africa, the United Kingdom or New Zealand. The number of years since graduation ranged from 5 to 43 years. All experiences occurred during domestic placements. Nine were completed in a metropolitan or regional area and two in rural areas. The majority of experiences were undertaken in hospital settings, (n = 12), followed by community (n = 3), and two placements were in institutions for individuals living with complex disabilities. See Table 1 for details of participants and their transformational experiences.

### 3.1 Similar but different

Through data analysis, the overarching theme identified was *similar but different*. No two transformational experiences were the same, ranging from the ordinary to the extraordinary. Experiences occurred within a variety of practice settings and participants received varying levels of support. The nature of the transformational experiences was shaped by the unique interaction between the individual characteristics of the participant, and the placement environment. However, despite their diverse nature, they were similar in that they displayed the same four key elements: they were challenging, emotionally laden, took time to process and had long‐term impacts.

#### 3.1.1 Challenges

Precipitating all transformational experiences was a challenge of some kind which included an issue related to either professional competence; perception of the profession; professional ideology; values, beliefs and understanding of the world; or a sense of acceptance.

Many participants' transformational experience involved a challenge either to their own competence or that of other health professionals. Working in multidisciplinary teams, two participants were faced with decisions being made by other professionals that were unsafe for their client. Participants' views of occupational therapy were also challenged and focused on their understanding of the occupational therapy role and of the profession's underlying values. For Hayden (bullied by his practice educators), his view of the profession as one that was caring was challenged. However, it was ‘… the fact the profession [in his case, the occupational therapy staff at his university] then rallied and said no, this is achievable [that made his experience transformational]’. The values he believed were central to the profession he was entering, were challenged but then reaffirmed.

Participants' experiences also challenged their personal values and understanding of the world. For Isla (saw a person with a severe disability for the first time), her experience evoked ethical questions such as ‘How does this happen?’, ‘Why isn't this better?’, and it ‘… brought up a lot of questions about rights … and caring for people who didn't have the ability to care for themselves’. However, some experiences affirmed participants' beliefs. Justine's experience (taking initiative for her own learning) confirmed her approach to life that ‘it pays to not rely on someone else …. It pays to want to have that internal drive’.

Participants' professional ideologies were challenged, and often, these came from something their practice educators said. Delia questioned the meaningfulness of her clients participating in the pottery group considering they were typically working‐class men.

How is this authentic like to these men? Would they really be going back to their houses and be doing pottery and clay? And the significant thing for me … I can't remember the exact words, it was something along the lines of, ‘It's just to keep them busy, it's just to stop these thoughts in their head.’ And just this moment kind of came to me and I was like … this is not, this is not occupational therapy for me.

Finally, participants' sense of acceptance was challenged. Amahle's past experiences as an outsider caused by prejudices held by others during her study, was dismantled by her practice educator. Whereas Carly's experience (being bullied by her practice educator) perpetuated her feeling of being an outsider, as her practice educator commented repeatedly about where she was from, her age and her hair colour. For Ellie (given responsibility for setting up a clinic), her practice educators exceeded the level of acceptance she was expecting as a student. Ellie spoke about how the responsibility of her project and being designated space within the clinic led her to feel like a valued part of the team.

#### 3.1.2 Emotionally laden

All transformational experiences were emotionally laden for the participants. Experiences were often so emotional that even many years after the event, participants were able to recall their feelings at the time. For some, just describing the experience in the interviews, many years after the placement, caused an emotional response. While a spectrum of emotions was experienced at the time of the event, many of them were negative and intense.

Hayden, who was bullied by his practice educator, reflected that his experience was ‘hard and terrible and awful at times’, and that what he experienced was ‘certainly emotional trauma’. Isla (saw a person with a severe disability for the first time) spoke about feeling ‘dreadfully sad’ for the girl she met and how it was ‘confronting’, ‘shocking’ and ‘scary as a student to see someone with such an enormous physical disability’ for the first time. Isla's experience occurred many decades ago, however ‘even now, it makes [her] feel sick’ to think of the stress placed on the girl's body. Chris, who failed his placement, described ‘a huge feeling of emptiness’ and loss as he received his final placement marks. Some participants also described physical and cognitive manifestations of their emotions at the time, such as being reduced to tears, panic attacks, word finding and memory difficulties, and a shattered tooth due to teeth grinding.

However, there were some positive emotions. For example, Ellie (given responsibility for setting up a clinic) stated that she felt proud, and a sense of satisfaction and achievement. For Justine (took initiative for her own learning), she was ‘happy with herself and what [she] did’ to drive her own learning. Georgia, who used professional judgement to advocate for her client, was ‘happy to stand [her] ground’ against a registrar wanting to unsafely discharge the client.

#### 3.1.3 Time to process

A number of participants spoke about being unable to make sense of the experience at the time. Many stated that they required time and reflection to understand and process the experience and for some this took years. Other participants spoke about requiring the help of others to make sense of their experience. Some received this help from practice educators and their peers, others did not access any support and required time and/or reflection to process their experience. This was illustrated by Chris (failed his placement) who stated, ‘Time was what helped. To be honest, I think I could've [sped] up that time if I'd been able to have a real conversation around that …’ experience to help contextualise it. And lastly, reflections were triggered by specific events unique to the individual and experience. For example, Carly (bullied by her practice educator) described that further insights came to her ‘when [she] supervised [her] first ever students, and just how it naturally came to [her] to run a placement very differently.’ Amahle, whose practice educator validated her lived experience as a person of colour, continues to reflect on her experience whenever she has been part of discussions about diversity within the profession.

A number of participants appeared to be still processing their transformational experience. Lingering emotions surrounding Carly's experience (being bullied by her practice educator) were still present, as she ‘very much still has that feeling of this is all my fault.’ Some still questioned what their practice educators were trying to communicate in the moment. Others stated that they continue to reflect actively on their experience.

#### 3.1.4 Long‐term impacts

All participants spoke about how their transformational experience taught them important lessons that have had long‐term impacts on their practice, understanding of the profession, and their personal development. These learnings led to a sense of gratitude for the experience, despite how it felt at the time.

The main long‐term impact participants experienced was in their professional practice. Almost all participants stated that their experience impacted the way they practised as an educator and how they supervised students and junior staff: ‘probably the biggest impact has been on when I have been supervising students … the power differential [has] … stayed with me’ (Belinda, working with a man to put his shoes and worrying she was doing it incorrectly). For example, some participants stated they adopted supervision techniques they found beneficial during their experience. Frankie (whose practice educator asked her what she was interested in observing) realised during her interview she had inadvertently mirrored the collaborative approach her practice educator used with her. Conversely, many participants stated that their supervisory approach was guided by providing the opposite of what they received during their experience or by providing what they wished they had received from their practice educators: ‘that placement … completely changed the way that I am as a practice educator, in the fact that I do almost everything different to what happened there’ (Chris, who failed his placement). Many participants spoke specifically about being mindful of the powerful position they hold within the student/practice educator relationship, no matter how hard they try to equalise it.

Participants also reported impacts on how they interacted with colleagues and clients. With colleagues, examples included advocating for occupation‐centred practice in the workplace or practicing cultural sensitivity when supporting junior staff. With clients, lessons included a deeper understanding of clients' experience with failure, having the humility to recognise clients were the experts in their health, and prioritising the client relationship regardless of the setting. The skills and lessons learned were reflective of participants' unique experiences.

The majority of participants stated that their experience specifically influenced their decisions regarding what practice setting, role and type of workplace they chose to work in following graduation. Four participants chose to work in mental health following their experience. Three participants stated this was due to avoiding the practice setting where their negative experience was: ‘I kind of knew at the back of my mind by the time I graduated that mental health would be my thing’ (Belinda, who was asked to ‘hose down’ a client), and the other was approaching mental health practice differently to how they witnessed it as a student. For Isla, meeting a young girl who was unable to support her head in a seated position inspired her to enter paediatrics and set up a seating clinic. Transformational experiences also prompted participants to consider the characteristics of future workplaces, including roles, workplace culture and whether occupational therapy was valued within the organisation. Carly (bullied by her practice educator) said her experience is responsible for her becoming a practice educator early in her career: ‘When I started this job I said to my boss, ‘I want to be a clinical educator so one less student has to go through what I went through.”

Participants developed a deeper understanding of the occupational therapy profession and role because of their experiences. This involved an understanding of the profession's scope of practice, potential practice settings and place within the health field. For example, Belinda (working with a man to put his shoes on) was able to recognise the significant impact occupational therapy could have on people: ‘the intensity of the emotion made it stick, the positive feedback … and why it was occupational therapy’. Three participants stated their experience confirmed that occupational therapy was their desired profession, including Frankie (whose practice educator arranged for her to observe a large number of other occupational therapists in the placement setting) who was initially studying occupational therapy to transfer into physiotherapy but decided to stay in occupational therapy.

Several participants discussed the personal growth their experience provided. Participants experienced growth in confidence, self‐worth, communication and self‐reflection, which were closely aligned to the professional impacts that occurred. Participants stated their experience was unenjoyable at the time, and others still viewed the experience with sadness, disappointment and regret. On balance, however, participants were grateful they underwent the transformational experience.

## 4 DISCUSSION

This study aimed to explore the transformational placement experiences that occupational therapists underwent when they were students, and the long‐term impacts of those experiences. The features of transformational experiences identified in this study align with features of the experiential learning process. Experiential learning is a continuous learning process where individuals continually have experiences, reflect on those experiences, conceptualise new learning and apply new ideas, creating knowledge through the transformation of experience (Kolb, 2015; Kolb & Fry, 1975).

No two experiences of the participants were exactly the same, and their impacts were unique to the individual and the experience. Participants who appeared to have similar experiences at face value in that they were negative (e.g., failing a placement and being bullied by a practice educator), found them transformational in different ways and for different reasons due to the uniqueness of each individual. This was acknowledged by some participants who articulated that their experience may not have been transformational for someone else. Experiential learning theorist Kolb (2015, chapter 4, para. 3) stated that ‘the learning process is not identical for all human beings,’ and is highly variable, complex and individual.

Kolb (2015) suggested that the learning process begins with an unsettling, unfamiliar or complex event, and the experiences of participants in this research is consistent with that suggestion: all participants' experiences began with a challenge. The challenges that prompted transformational experiences included singular events on placement or an entire placement, as well as significant confronting events or what might appear to be inconsequential events. To date, the literature documenting what we have termed transformational experiences describes experiences that are clearly potentially impactful, such as the death of a client (Warne & Hoppes, 2009), workplace violence (Gair & Thomas, 2008), and completing a rural or international placement (Barker et al., 2010; Crawford et al., 2017; Davies et al., 2017; Haro et al., 2014; Markewitz, 1992; Sim & Mackenzie, 2016; van Rensburg & Du Toit, 2016). As such, a finding in this research that seems novel, is that events that might be seen as ordinary (e.g., supporting a client to put on shoes or completing a showering assessment/intervention) could also be transformational.

Intense emotions also appear inextricably linked to transformational experiences. Vogel and Schwabe (2016), who investigated the impact of stress on learning, explain that emotional material is better remembered than neutral material. Emotions, particularly positive ones, can enhance learning (Vogel & Schwabe, 2016; Zull, 2002). Whereas negative or threatening experiences can make it more difficult for individuals to focus and absorb the experience fully (Zull, 2002). This was not the experience of participants in this study. Participants were able to recall their transformational experiences in quite some detail. Vogel and Schwabe (2016) acknowledged that despite research establishing that stress changes learning and memory processes, it is still unclear whether differences in personality and gender can moderate how stress effects learning. Further research may clarify whether personality plays a role in experiential learning as described by Kolb's (2015) and if this changes under stress as proposed by Vogel and Schwabe (2016).

The second stage of the experiential learning process involves reflection (Kolb, 2015). The fact that some participants were still processing their experiences and came to new understandings during the interviews, demonstrates the iterative nature of the experiential learning process. Participants continued to learn from their transformational experience as they gained new knowledge and life experiences. This study supported findings from Mann et al. (2009), which identified that time, perspective and the help of practice educators were beneficial for reflection. Mann et al.’s (2009) systematic review into reflective practice also identified peer support, a safe atmosphere and a facilitating context were other factors associated with successful reflection. Due to the cyclical reflective process continually integrating new knowledge, experiential learning is lifelong. Findings of this study show that processing of transformational experiences can continue for many years after the event.

The findings of this study regarding the impacts of transformational experiences expand on those identified by Lew et al. (2007). This study identified that participants: experienced increased resilience and self‐reliance; choose an area of practice different to that of their negative placement experience; and that their supervisory approach was shaped by the supervision they received (specifically, doing the opposite). The choice of participants to work in practice settings in which they had positive experiences and avoid settings they had negative experiences as a student, has been a long‐established short‐term impact (Chiang et al., 2013; Christie et al., 1985; Crowe & Mackenzie, 2002; Keller & Wilson, 2011). This study adds to that by demonstrating that this impact is long‐lasting. However, one participant did report entering the same practice setting that their negative experience occurred in and decided to approach it differently to how they experienced as a student. This is an example of the experimental stage of experiential learning, as participants use the knowledge gained from reflection and apply what they have learned to the world (Kolb, 2015).

Lastly, findings demonstrate the significant role practice educators have in determining if a transformational experience will be positive or negative and so add to the existing literature (Gair & Thomas, 2008; Lew et al., 2007). WFOT (2016) states that placement supervision should be appropriate to the students' knowledge base and learning needs. Although some of these placements took place before the 2016 standards were implemented, they codified what has been long recognised as best practice (e.g., Kilminster & Jolly, 2000), that is, to ensure that expectations and responsibilities should be known, clear, and explicit, so that all parties are adequately prepared and supported fulfil their roles (WFOT, 2016). Whilst participants were able to learn from their experiences, some of them should not have occurred, for example, bullying. This research demonstrates the importance of ongoing education for practice educators regarding expectations and responsibilities, along with support to understand the potential impact of placement experiences on students, well beyond placement. Indeed, all participants in practice education need to be cognisant of the range of experiences that can occur on placement and the impact they can have on students' during their education (Grant et al., 2022) but also longer‐term after graduation.

## 5 LIMITATIONS

Some participants provided experiences that could be considered expected placement learning instead of transformational experience, for example, a broadened understanding of the occupational therapy role and profession. Despite written and verbal descriptions, and because the term ‘transformational experiences’ was used in a new way for this research, it is possible participants misunderstood the term. While recruitment strategies aimed to provide a broad range of participants, it is noted that eight (62%) participants were educated in Australia. Furthermore, all but one experience occurred in Western countries and the majority occurred in a hospital setting. A broader range of perspectives would be important in improving the generalisability of the findings.

## 6 IMPLICATIONS FOR FUTURE RESEARCH

Future research into the role of stress and personality types may benefit practice educators and universities to tailor support for students on placement and assist in maximising the positive impacts of transformational experiences. Further research into long‐term impacts should be conducted to establish whether (and which) short‐term impacts remain long‐term or change over time. Any future research might benefit by focusing on specific types of practice settings (e.g., intercultural, hospital‐based, mental health etc.) and/or student demographics (e.g., age, cultural background, gender and disability status) to identify whether impacts are generalised or associated with particular settings or populations.

## AUTHOR CONTRIBUTIONS

All authors made substantial contributions to this study. All authors had input into the conceptualisation and development of the study. All authors completed data analysis. O.N. developed the manuscript, and all authors were involved in the revising of this paper. All authors gave final approval for the article to be submitted.

## CONFLICT OF INTEREST STATEMENT

No conflicts of interest to declare.

## Data Availability

Research data are not shared.

## Appendix A INTERVIEW GUIDE

Demographic Information

1. Where did you graduate from? This information may be useful during analysis but will be deidentified or generalised if it is relevant to publish.
2. When did you graduate?
3. How long have you worked as an occupational therapist?
4. What practice settings have you worked in as an occupational therapist?

Question 1:

Can you tell us about your transformational experience?

Prompt: How long ago was the experience that you are talking about today?

Question 2:

What long term impact did this transformational experience have on you?

Prompts:

- At the time
- Since the event
- Long term
  b Personally
    - Physical?
    - Cognitively?
    - Socially?
    - Emotionally?
    - Spiritually?
  c Professionally
    - Job choice?
    - Whether or not participants took on students?
    - How they supervised their students as practice educators?
    - Level of career satisfaction?
    - Relationships with colleagues?
- Temporal
  b What was the impact at the time? (Impact at the time)
  c How long was the impact felt for? (Duration of impact)
  d Is this impact still felt today? (Impact still felt today)
  e Has this impact changed over time? How?

Question 3:

Why do you think this experience has been transformational?

Question 4:

Are you grateful or regretful this experience happened?

Prompts:

- Given the choice, would you rather have gone through the experience or not?
- How do you think your life would be if you did not have this transformational experience?
- Has this changed over time?

Question 5:

Is there anything we have not asked you that you think is important for us to know?
